# Teaching ultrasound in a curricular course according to certified EFSUMB standards during undergraduate medical education: a prospective study

**DOI:** 10.1186/1472-6920-13-84

**Published:** 2013-06-11

**Authors:** Hauke S Heinzow, Hendrik Friederichs, Philipp Lenz, Andre Schmedt, Jan C Becker, Karin Hengst, Bernhard Marschall, Dirk Domagk

**Affiliations:** 1 1Department of Medicine B, University Hospital Muenster, Albert-Schweitzer-Campus 1, A1, 48149, Münster, Germany; 2 Institute for Education and Student Affairs (IfAS), University of Muenster, Muenster, Germany

**Keywords:** Undergraduate medical education, Ultrasound, Ultrasonography, Clinical competence

## Abstract

**Background:**

As a non-invasive and readily available diagnostic tool, ultrasound is one of the most important imaging techniques in medicine. Ultrasound is usually trained during residency preferable according to German Society of Ultrasound in Medicine (DEGUM) standards. Our curriculum calls for undergraduate training in ultrasound of medical students in their 4th year of undergraduate education. An explorative pilot study evaluated the acceptance of this teaching method, and compared it to other practical activities in medical education at Muenster University.

**Methods:**

240 medical students in their 4th year of undergraduate medical education participated in the training and completed a pre- and post-questionnaire for self-assessment of technical knowledge, self-assurance of the procedure, and motivation in performing ultrasound using a Likert scale. Moreover, students were asked about their interest in pursuing a career in internal medicine. To compare this training to other educational activities a standardized online evaluation tool was used. A direct observation of procedural skills assessment (DOPS) for the first time applied on ultrasound aimed to independently assess the success of our teaching method.

**Results:**

There was a significant increase in technical knowledge and self-assurance (p < 0.001) of the students’ self-assessments. The clinical relevance and self-motivation of the teaching were evaluated positively. The students’ DOPS results demonstrated proficiency in the understanding of anatomic structures shown in ultrasonographic images, including terminology, machine settings, and transducer frequencies.

**Conclusions:**

Training ultrasound according to certified DEGUM standards was successful and should be offered in undergraduate medical education. The evaluation of the course affirmed the necessity, quality and clinical relevance of the course with a top ranking score of hands-on training courses within the educational activities of the Medical Faculty of Muenster.

## Background

### Relevance of ultrasound

Ultrasound is the most often used imaging tool in clinical practice [1]. Unlike computed tomography and magnetic resonance imaging, the technique is portable and quick. Ultrasound can be used to guide interventional procedures and is the least invasive imaging modality available. Additionally, it allows to combine anamnestic findings, clinical examination and imaging in a short period of time [2].

After completion of medical school, young interns are already expected to have fundamental theoretical and practical skills in ultrasound since these are already an integral part of clinical practice. Compared to other imaging modalities that work with standardized planes, sonographic findings are mainly dependant on the investigator‘s technical skills. Thus, in addition to understanding the physical principles, practical experience with the equipment of ultrasound devices is required for qualified diagnostic statements.

So far ultrasound has only been incorporated into undergraduate medical student curricula only to a limited degree [3-8] and has not been systematically implemented as a curricular course to be learned by every student in undergraduate medical teaching [3]. Lately, numerous study groups have described their efforts to integrate ultrasound into a medical curriculum demonstrating the increased awareness of the relevance in undergraduate medical education [3,7,9-12].

The intention of our initiative hands-on training of ultrasound is to allow each student of Muenster medical school to gain individually skills in imaging various human organs and its pathologies, creating an individual foundation for further medical practice, according to DEGUM sonographic guidelines for undergraduate medical students [13].

With our study we aim to assess whether curricular ultrasound education according to the DEGUM ultrasound guidelines for undergraduate medical students is feasible and valuable. The study‘s objectives are assessed by a self-assessment questionnaire to determine the students‘ opinion of their technical knowledge, self-assurance and motivation to perform the examination, as well as the safety of the procedure. In order to monitor each student‘s improvement in performance, a practical assessment tool (DOPS) was applied for the first time on ultrasound at the end of the course.

## Methods

The study was carried out during 2010 and 2011 at the Medical Faculty of Muenster. 240 students in their 4th year of undergraduate medical education participated in the study.

### Course model and curricular content

The course model was designed as follows:

The complete curriculum consisted of 28 hours over 9 weeks. The practical training encompassed 20 hours, consisting of ten sessions each lasting two hours. In groups of five, students worked on high-end ultrasound devices (Hitachi EUB 5500 HV). The sessions were accompanied by weekly lectures focusing on nine different session themes addressing the basics of ultrasound techniques, physics and tools, abdominal and pelvic anatomy, thyroid gland, Focused Assessment with Ultrasound for Trauma (FAST) and general pathologies.

Table 1 gives an overview of the ultrasound curriculum at Muenster University.

**Table 1:** University of muenster medical faculty ultrasound curriculum

**Session**	**Objectives**
*I. Introduction of ultrasound*	Ultrasound Principles
	Transducer Types
	Handling of the ultrasound device
	Knowledge of standardized sonographic planes
*II. Liver*	Demonstration of normal findings
	Volumetry of the liver
	Assessment of fatty liver
	Assessment of suspicious focal lesions
	Doppler analysis of the portal vein and intrahepatic blood flow
*III. Portal area of liver*	Demonstration of normal findings
	Cholestasis
	Measurement of the gallbladder wall
	Cholecysto/docholithiasis
	Volumetry of the gallbladder
	Polyps of the gallbladder
*IV. Kidney*	Demonstration of normal findings
	Volumetry of the kidneys
	Parenchym/pyelon relation
	Assessment of hydronephrosis
	Adrenal gland
*V. Pancreas*	Demonstration of normal findings
	Assessment of fatty pancreas disease
	Assessment of suspicious focal lesions
	Assessment of pancreatitis
*VI. Splen*	Demonstration of normal findings
	Volumetry of the splen
	Assessment of suspicious focal lesions
*VII. Retroperitoneum*	Demonstration of normal findings
	Assessment of lymphadenopathy
*VIII. Thyroid gland*	Demonstration of normal findings
	Volumetry
	Assessment of suspicious focal lesions
	Assessment of different types of thyreoiditis
	Doppler analysis of cervical vascularity
	Parathyroid
*IX. FAST*	Demonstration of normal findings
	Ultrasound in emergency care
	Assessment of pneumothorax
	Assessment of pleural effusion
	Assessment of intra-abdominal free fluid
	Assessment of pericardial effusion

Lecture notes for each session were developed describing the educational objective, including specifics on scanning techniques and patient positioning, general anatomy and main pathological findings.

In the course multiple teaching modalities were applied. These consisted of live-demonstrations during each lecture of interesting sonographic findings on patients after written or oral informed consent had been obtained. Moreover, ultrasound scanning techniques were efficiently presented during lectures on human models. During lectures we also demonstrated video clips and still images of general pathological sonographic findings. In addition, a series of web-based learning modules (e.g. step by step module on measuring the portal vein flow), web-based case studies with video clips and frames as well as web-based questions to assess the student’s knowledge of ultrasound and its clinical application for each session theme were applied. The following web-based learning modules were applied.

•Knobology

•Demonstration of standardized sonographic planes

•Measurement of portal vein flow

•Sizing of organs

•Volumetry of gall bladder and thyroid gland

•Sonographically-visualised collapse of the inferior vena cava ( IVC)

•Demonstration of a standardized sonographic investigation of the abdomen

•Case report on liver cirrhosis

•Case report on choledochocystolithiasis

•Case report on nephrolithiasis

•Case report on retroperitoneal lymphadenopathie

•Case report on acute and chronic pancreatitis

•Case report on splenic infarction

•Case report on hyperthyreosis

•Case report on right ventricular dilation

•Differential diagnosis of liver and pancreatic lesions

•Pathologic findings of kidney, spleen, gall bladder, biliary tract, vessels, lymphnodes, intestine and thyroid gland

The hands-on sessions were supervised by board certified DEGUM level 2 to 3 sonographic experts.

### Clerkship

During an internal medicine clerkship as a clinical correlation component after taking the curricular ultrasound class students are additionally exposed to in- and outpatients in hands-on training for 4 hours in our ultrasound unit practising the acquired scanning skills under guidance and supervision by board certified DEGUM level 2 to 3 sonographic experts.

### Direct observation of procedural skills

**Figure 1:**
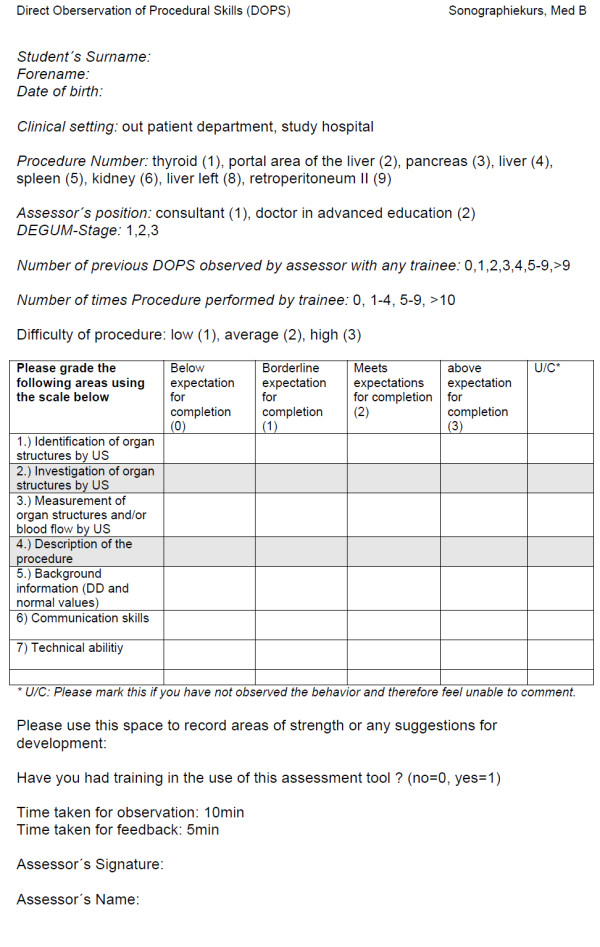
Direct observation of procedural skills assessment form.

DOPS is a practical assessment method assessing the procedural skills of trainees in a workplace setting [14]. Using a 6-point rating scale (0–1 below the expected level of competency, 2 reflecting a borderline level of competency, 3 meeting the expected level of competency and 4–5 representing an above expected level of competency) the trainee’s performance is evaluated during 15 minutes of observation time [15]. DOPS is both formative and summative, thus about 5 minutes are dedicated to direct feedback to the students [14]. In alignment with the described method by Wragg and colleagues focusing on a list of commonly performed procedures such as endotracheal intubation, nasogastric tube insertion, arterial blood sampling, we have modified the assessment method and adapted to the performed procedure ultrasound (Figure 1). The DOPS method requires an assessor to independently grade a trainee each on one practice item. Assessments were held in the study hospital Muenster (Studienhospital Münster®) in an authentic workplace environment. We cannot completely rule out, that the assessors evaluated occasionally candidates who they had recently taught or trained during the course, thus leaving space for assessment bias.

### Development process of DOPS items and criteria

The DOPS assessment was developed by an expert group composed of a gastroenterologist (D.D), two doctors of general medicine (P.L, A.S.), a registrar in gastroenterology (H.H), a surgeon (B.M) and clinical educationalists (H.F., J.B.) via a series of repetitions. Eight different practice items were developed and randomly and blinded assigned to the assessors and trainees (thyroid (1), portal area of the liver (2), liver (3), pancreas (4), spleen (5), kidney (6), liver left (7), retroperitoneum (8). All DOPS assessments were combined with a simulated clinical case example. There were seven main domains, identification and investigation of organ structures, measurement of organ structures, description of the procedure, background information, communication skills and technical abilitiy. Grading of scanning skills for example was based on selecting the appropiate ultrasound probe, holding the probe; adjusting gain, depth and focal zones. These points are compromised in DOPS item 7 (Figure 1). DOPS item 2 and 3 assessed dynamic maneuvers as measuring the portal flow and adequate scanning of an organ. Item 5 “background information” included e.g. normal values of the common bile duct, portal vein flow, normal volumetry values of thyroid gland. As all DOPS assessment are combined with a simulated clinical case example naming of related information are also considered as background information. For further details of the DOPS criteria please contact the authors since the data is the topic of a consecutive study.

DOPS Assessors undertook the assessment for each organ themselves and afterwards an interactive workshop was held discussing point-by-point each item and comparing the individual gradings. Assessors then went on assessing 20 voluntary students. The DOPS gradings of each assessor were anonymously compared with data from other assessors and accessible for further feedback.

### Conceptual framework

As a conceptual framework for the curricular development of practical skills course models, the “Theory of Expertise” by Ericsson et al. is suitable [16-18].

According to this theory, a practical task, such as a “real-life performance” should motivate students, integrate their pre-existing knowledge, and offer opportunities to repeat. It should be accompanied by immediate feedback and be presented in various contexts [19].

### DEGUM Guidelines

The course concept was designed according to the widely accepted DEGUM sonographic guidelines for teaching of ultrasound [20] and was certified by the DEGUM committee‘s offices in April 2010. It conveys basic knowledge in physical and technical principles of ultrasound, in instrument adjustment, writing of diagnostic findings, and documentation to the students.

### Questionnaire

The acceptance by the students and the relevance for learning of this new teaching method was also scrutinised by a pre-post questionnaire (Table 2).

**Table 2:** Results of the pre-post questionnaire to various aspects of ultrasound

**Questionaire ultrasound-training in undergraduate medical education**				
**Statement**	**Pre-mean**	**Post-mean**	**Difference**	**Significance**
I have sufficient technical knowledge about the ultrasound examination	1.98	3.61	↓*, d=1.635	p<.001
I have enough self-assurance in the use of ultrasound	1.94	3.51	↓*, d=1.568	p<.001
I am motivated to perform the ultrasound examination in patients	4.69	4.66	(↓)	n.s.
I consider a further career in the speciality of internal medicine.	3.48	3.59	(↑)	n.s.
I enjoyed the training (in terms of positive stimulation)	-	3.99	-	-
The training improved my skills with respect to a better understanding of pathophysiology and anatomy of internal diseases	-	4.08	-	-

Level of agreement on a 5-point Likert-scale (strongly agree = 5, agree = 4, neither agree nor disagree = 3, disagree = 2, strongly disagree = 1).

The students recorded their gender, age and level of agreement on a 5-point Likert-scale (strongly agree = 5, agree = 4, neutral = 3, disagree = 2, strongly disagree = 1) with the listed statements that characterised their perception of the question. Because we obtained verbal informed consent for participation in the study from the participants, the local Ethics Committee of the university of Muenster waived requirements for an approval procedure.

### Statistical analysis

The collected data were analysed with the program SPSS® IBM® Statistics 19 (SPSS. Inc.). Means and standard deviations were calculated as descriptive parameters. Parametric tests were used to test the hypotheses [21]. The pre-post results were compared using paired t-tests or chi-square tests. Differences were considered statistically significant if p < 0.05.

### EVALuna evaluation

The online evaluation of each course via the EVALuna system (Binary Design GmbH, Muenster) is a requirement for the registration of exams at the Medical Faculty of Muenster.

EVALuna guarantees anonymous evaluation of each curricular course at the Medical Faculty of Muenster. Students are asked to evaluate the course on a visual analogue scale from 1 (“very good”) to 100 (“very poor”). In addition, there is the possibility to enter comments in free text form.

## Results

### Questionnaire

At the end of the course 192 of 240 ciphered questionnaires were available for analysis, which is an 80.0% (95%-CI 74.9-85.1%) return rate.

The average age of the students was 23.4 years (SD ± 1.3), 61.4% (95%-CI 54.6-68.3%) were female. According to our data, there was a significant increase in the self-assessed technical knowledge (d = 1.635, p < 0.001) and self-assurance (d = 1.568, p < 0.001) of the students in using ultrasound. The motivation (pre-mean 4.69, post-mean 4.66) and the perspective of a career in internal medicine showed no significant differences. The effectiveness (mean 4.08) and the positive stimulation (mean 3.99) of the teaching model were rated at the top area of the Likert-scale (Table 2).

### EVALuna

**Figure 2:**
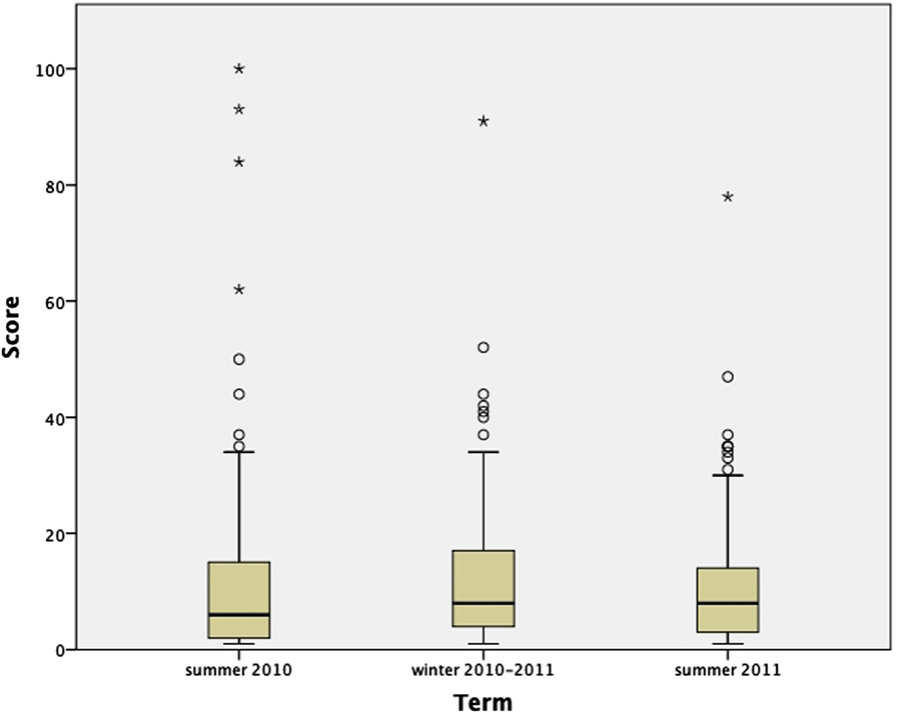
**Results of the evaluation by the students.** Boxplots of the EVALuna-visual analog scale between 1 (“very good”) and 100 (“very poor”) The top of the box represents the 75th percentile, the bottom of the box represents the 25th pecentile, and the line in the middle represents the 50th percentile. The whiskers (the lines that extend out the top and bottom of the box) represent the highest and lowest values that are not outliers or extreme values. Outliers (values that are between 1.5 and 3 times the interquartile range) and extreme values (values that are more than 3 times the interquartile range) are represented by circles and stars beyond the whiskers.

The EVALuna analysis showed similar results (Figure 2). 238 participants evaluated the course with an average feedback of 12.06 points (95%-CI 10.13-14.00) on a visual analogue scale from 1 (“very good”) to 100 (“very poor”).

The EVALuna free-text feedback indicated that the students agreed or strongly agreed that their experience with ultrasound education was positive and that ultrasound knowledge and training are useful to physicians. However, students criticized the group size of five to six students as too big with not enough time to perform ultrasound individually within the session time. They also requested additional appointed time for further consolidation of sonographic skills.

## DOPS -results

188 questionnaires of 193 assessments could be analysed after the course (return rate of 97.4%; 95%-CI 95.2-99.7%).

**Figure 3:**
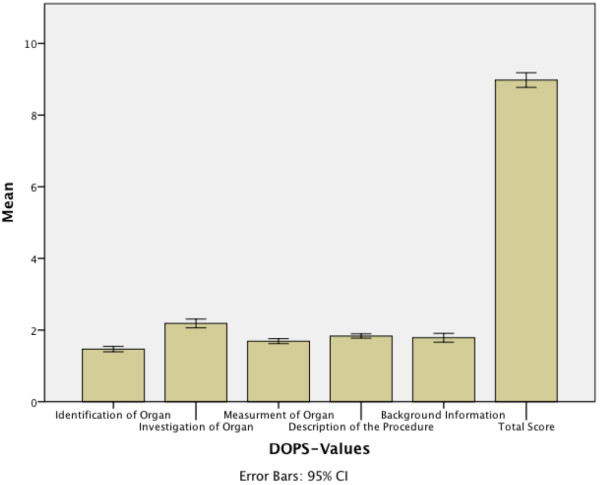
**Bar Charts of the DOPS-Scores.** Performance-values of the students in the “direct observation of procedural skills” (DOPS) at the end of the course.

Students had more difficulties in the identification of organs (mean 1,47; 95%-CI 1,39-1,54) than in the investigation of organs (2,19; 95%-CI 2,07-2,31) (Figure 3).

The measurement of organs (1,69; 95%-CI 1,62-1,76), description of procedure (1,84; 95%-CI 1,77-1,99) and the giving of background information (1,79; 95%-CI 1,66-1,91) met our expectations. The total score was 8,87 (95%-CI 8,63-9,11) with a maximum score of 12.

## Discussion

After completion of medical school, young interns are often expected to already have fundamental theoretical and practical skills in ultrasound, as these are an integral part of clinical practice [22].

There is broad agreement that medical educators should prepare students for further ultrasound education in residency (Arger PH et al.; Teaching medical students diagnostic sonography). Lately, numerous study groups have described their efforts to integrate ultrasound into a medical curriculum demonstrating the increased awareness of the relevance in undergraduate medical education [3,7,9,10,12,23,24].

Then again it is general consensus that medical education has to be carried out on real patients at a certain point in order to consolidate the acquired skills of students or doctors [25]. On the other hand, patients have to receive the best care, and their safety has to be ensured. The traditional practice of “see one, do one, teach one“is thus no longer adequately ethically justifiable [25] and the acceptance for this method has decreased markedly [26], raising a conflict between the quality of medical education and the quality of care and patient safety [25]. Simulation of practical skills can be helpful to balance these differences. Simulated trainings as implemented in our course setting at the study hospital Muenster enable students to learn ultrasound in a controlled and protected simulation setting almost identical to a future medical occupational environment. In this controlled learning environment, exercises can be practised successively to gain and guarantee adequate and effective experience [27] and students may optimise their knowledge and skills [28,29]. It has consistently been criticized, that medical schools are too theoretical and over-emphasise teaching knowledge-based content [30].

Simulation, as used in our ultrasound course, addresses these concerns. As the analysis of the questionnaire reflects, significant increase in the self-assessed technical knowledge (d = 1.635, p < 0.001) and students’ self-assurance (d = 1.568, p < 0.001) in using ultrasound was achieved. The motivation to perform ultrasound remained very high (pre-mean 4.69, post-mean 4.66). A sonographic course-model integrates previous acquired knowledge of anatomy and pathology in a clinical context. Teaching in small groups allows each student repetition, which can even be optimized by further group-size reduction, as requested in feedback from the students. Moreover, highly qualified board certified DEGUM sonographic experts provide quality professional feedback to the students. Thus, it is not surprising that the students’ evaluation confirmed excellence in our undergraduate ultrasound course model (Figure 2).

Analysis of the EVALuna free text feedback further strengthens this rationale. The students’ feedback has helped direct the development of the course and has facilitated a sense of student partnership in the curriculum.

As it could be gathered from the free text feedback, the group size of 5–6 students was criticized, thus leading to a decrease of students’ group size to three to four students during the current term. Moreover, the request for additional appointed time for further consolidation of sonographic skills was realized in form of supplemental voluntary open sessions.

The opinion of students who felt competent in imaging various abdominal organs and operating ultrasound devices effectively, could be confirmed in a practical assessment at the end of the term. In this way, assessment by DOPS is an important source of constructive feedback.

Hofer et al. recently designed an Objective Structured Clinical Examination-Course (OSCE) for ultrasound classes with acceptable reliability. Cronbach’s alpha reached values above 0.8 when more than 8 stations were combined in one course for ultrasound [31]. Thus, this ultrasound-OSCE needs a large number of stations with quite long test time, which usually exceeds the resources available at faculty.

Further development of structured clinical and workplace-based assessments, such as DOPS, address these limitations. DOPS has been evaluated to be a reliable and valid formative assessment tool [32].

Moreover, a study by Barton and colleagues has reliably assessed the skills of endoscopists to certify competence using DOPS [33]. It is a method of assessment developed specifically for the assessment of practical skills [14].

Additionally, the use of formative feedback may address further domains of professional behaviours, attitudes and communication in order to develop expertise in the assessed procedure. Hence, it was self-evident to apply a modified DOPS (Figure 1) on our curricular ultrasound course.

Analysis of the DOPS results demonstrated proficiency in the students’ understanding of anatomic structures shown in ultrasonographic images, including terminology, machine settings, and transducer frequencies, as displayed in Figure 3.

## Conclusion

A previous study by Yoo *et al*. [34] has suggested that undergraduate medical students can obtain a resident-level understanding of ultrasound provided that they are given proper means and methods of training. Our prospective pilot study demonstrates the educational benefits of a curricular ultrasound course according to the DEGUM guidelines for an undergraduate medical institution. Our findings indicate that simulated training in a highly professional undergraduate setting is possible and can be integrated in a curricular theme in medical school education, and further, is a positive way to increase physician expertise with diagnostic imaging technology and improve the quality of patient care [35].

The students’ self-assessed technical knowledge as well as their self-assurance in the use of ultrasound was enhanced. The motivation to perform ultrasound, the effectiveness, and the enjoyment of this training model are improved. The application of the educational method of simulation to ultrasound has made this one of the top ranking courses within the educational activities of our Medical Faculty. Admittedly, the experience and the results of our curriculum are not generalizable and it is unlikely that there will be a curriculum for ultrasound that will fit any medical school around the globe. Modifications will definitely be necessary based on faculty experience and expertise in ultrasound, available technical resources and support [3]. However, we think that our report adds valuable information to the ongoing debate of ultrasound curricula in undergraduate medical education.

## Competing interests

The authors declare that they have no competing interests.

## Authors’ contributions

HH set up the course, conceived of the study, coordinated the study and drafted the manuscript. HF conceived of the study, participated in its design and coordination, performed the statistical analysis and drafted the manuscript. . DD set up the course, participated in the coordination and design of the study and helped to draft the manuscript. JCB, BM, KH, AS and PL participated in its design and helped to draft the manuscript. All authors read and approved the final manuscript.

## Pre-publication history

The pre-publication history for this paper can be accessed here:

http://www.biomedcentral.com/1472-6920/13/84/prepub
